# Case Report: Type II Bartter syndrome with a novel *KCNJ1* variant in a premature neonate presenting with features of salt-wasting congenital adrenal crisis and pseudo-hypoaldosteronism

**DOI:** 10.3389/fped.2025.1550608

**Published:** 2025-06-24

**Authors:** Heung-Ching Tsui, Hua Tse-Timothy Cheng, Kai-Yee Lam, Lai-Ting Leung, Ka-Nam Au, Wai-Yu Wong, Luen Yee-Sylvia Siu, Lap-Ming Wong

**Affiliations:** ^1^ Department of Pediatrics and Adolescent Medicine, Pamela Youde Nethersole Eastern Hospital, Hong Kong, Hong Kong SAR, China; ^2^ Department of Pathology, Hong Kong Children’s Hospital, Kowloon, Hong Kong SAR, China; ^3^ Department of Clinical Genetics, Hong Kong Children’s Hospital, Kowloon, Hong Kong SAR, China; ^4^ Department of Pediatrics and Adolescent Medicine, Tuen Mun Hospital, Hong Kong, Hong Kong SAR, China

**Keywords:** antenatal Bartter syndrome, pseudohypoaldosteronism, adrenal crisis, genotype phenotype correlation, KCNJ1 gene, severe polyhydramnios

## Abstract

**Introduction:**

Bartter syndrome (BS) is a rare group of inherited renal tubulopathies. Diagnosis of BS type II is challenging in the neonatal period as its clinical findings and biochemical features may mimic that of adrenal crisis and pseudo-hypoaldosteronism (PHA) initially. Treatment should be instituted immediately for acute adrenal insufficiency as it is a medical emergency, then modified according to available investigation results and treatment response.

**Case presentation:**

We describe a premature female neonate with an antenatal history of severe unexplained polyhydramnios, presented with features of adrenal crisis managed with hydrocortisone and fludrocortisone. Initial endocrine investigations excluded salt-wasting congenital adrenal hyperplasia (SW-CAH) and pointed to the diagnosis of PHA with hyperreninemic hyperaldosteronism. Hydrocortisone was gradually weaned off while fludrocortisone was continued for sodium retention effect. Hyperkalemia quickly transited into hypokalemia requiring high potassium requirement. Clinical and biochemical features of BS gradually evolved with polyuria, excessive weight loss, hypochloremic metabolic alkalosis and hypercalciuria at 1 week of age. Urgent trio whole exome sequencing (WES) subsequently confirmed the diagnosis of BS type II where compound heterozygous missense variants were identified in the *KCNJ1* gene, one of which was a novel variant. Fludrocortisone was stopped and indomethacin was started with favorable outcomes.

**Conclusion:**

Though hypokalemia is the key feature of BS, transient hyperkalemia can occur in the early neonatal period in BS type II. Antenatal history should be enquired thoroughly to look for presence of severe unexplained polyhydramnios. The diagnosis of BS type II should be considered if other biochemical features are present. Genetic tests are important to provide a definite diagnosis and guide subsequent management and genetic counselling.

## 1 Introduction

Bartter syndrome (BS) is a group of inherited salt-losing renal tubulopathies characterized by impaired salt reabsorption in the thick ascending limb (TAL) of the loop of Henle with activation of the renin-angiotensin system, resulting in polyuria, hypokalemia, hypochloremic metabolic alkalosis and normotensive hyperreninemic hyperaldosteronism (1).

Acute adrenal insufficiency is rare in the neonatal period and salt-wasting congenital adrenal hyperplasia (SW-CAH) is the commonest cause of it (2). Adrenal crisis is suspected when newborns present with hyperkalemia with hypotension. Life-saving treatment of adrenal crisis should be initiated immediately. In contrast to other types of BS, BS type II may present with transient hyperkalemia in the neonatal period, mimicking SW-CAH and pseudo-hypoaldosteronism (PHA) type I until urine tests and serum renin and aldosterone are available. Genetic study should be done early to get a definitive diagnosis and guide the subsequent management. Before the genetic result is available, treatments should be modified according to the contemporaneous biochemical results and clinical progress.

In this case report, we describe a premature female neonate presented with clinical features of acute adrenal insufficiency. The clinical condition and biochemical parameters were stabilized after the initiation of treatment for adrenal crisis. The patient developed polyuria and hypokalemia at 1 week of age. Initial endocrine investigations excluded SW-CAH and pointed to the diagnosis of PHA type I with markedly elevated serum renin and aldosterone. However, the urine potassium was suggestive of salt losing which should not be present in PHA type I. Genetic test confirmed the diagnosis of BS type II with compound heterozygous likely pathogenic variants identified in the *KCNJ1* gene. The management approach was then refined according to the genetic results.

## 2 Case presentation

### 2.1 General information

A baby girl was delivered at gestation 29 + 4 weeks by crash Caesarean section due to fetal distress with a birth weight of 1.27 kg which was appropriate to gestational age. Apgar score was 6 at 1 min and 7 at 5 min of life.

This was the first pregnancy of a non-consanguineous Chinese couple. Mother had seronegative rheumatoid arthritis with stable control on sulphasalazine. There was no family history of endocrine or renal diseases. Antenatal ultrasonography revealed significant polyhydramnios since 24 weeks of gestation. Amniotic Fluid Index (AFI) was 47.5 cm with a normal stomach bubble. There was no gestational diabetes mellitus and the morphology scan was unremarkable. Amnioreduction was performed at 25 weeks of gestation with antenatal steroid given. Chromosome microarray on the amniotic fluid was normal.

After delivery, the baby required transient invasive ventilatory support. Neonatal sepsis workup was negative. There was hypotension requiring saline boluses and dopamine infusion for less than 1 day. Newborn examination showed no dysmorphism and she had normal female genitalia with no signs of virilization.

### 2.2 Diagnostic assessment

**Figure 1:**
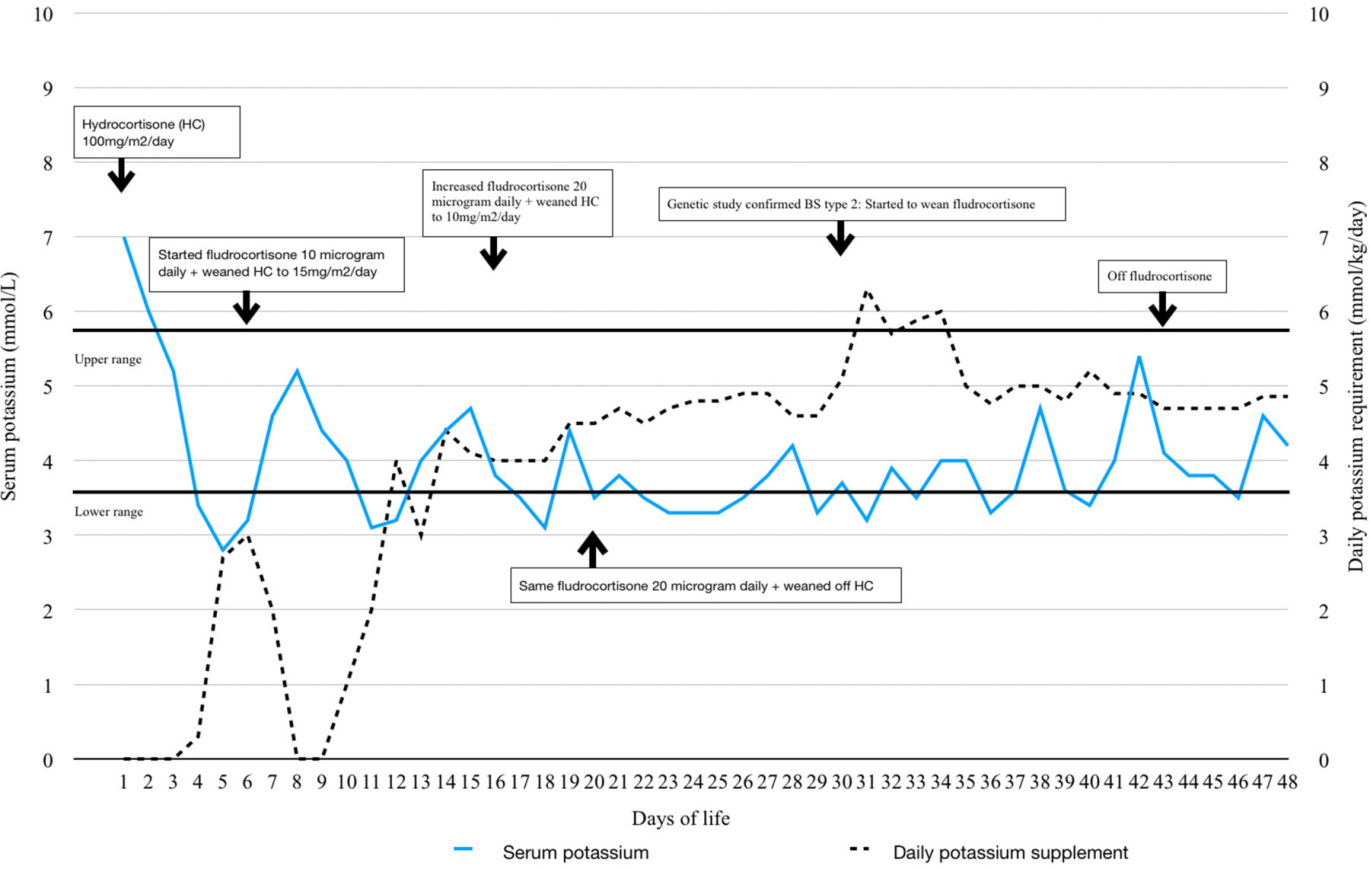
Trend of serum potassium, daily potassium supplement, hydrocortisone and fludrocortisone dosage.

At 20 h of life, routine blood test showed severe hyperkalemia with serum potassium level of 7 mmol/L (reference range 3.7–5.9 mmol/L) and hyponatremia with serum sodium level of 131 mmol/L (reference range 133–146 mmol/L) (Figure 1). Serum urea was raised at 10.9 mmol/L (reference range 1–8.2 mmol/L). Serum creatinine was normal. Plasma glucose was 2.9 mmol/L. There was no significant metabolic acidosis nor alkalosis with gas showing pH 7.4, bicarbonate 19.3 mmol/L (reference range 22–26 mmol/L) and base excess −4 mmol/L (reference range −2 to −3 mmol/L). There was mild tachycardia with heart rate 180/min. Blood pressure was normal.

The presumptive diagnosis was acute adrenal insufficiency. Endocrine investigations including cortisol, adrenocorticotropic hormone (ACTH), 17-hydroxyprogesterone (17-OHP) and androgen profile were performed. Then, she was treated immediately with fluid resuscitation and intravenous hydrocortisone at 100 mg/m^2^/day and was put on fludrocortisone later. Dextrose-insulin drip was given for 1 day and her hyperkalemia promptly settled (Figure 1). She also had polyuria with urine output up to 8 ml/kg/h since day 1 of life with significant weight loss of 20%. Despite hyperkalemia, the urine potassium was 5 mmol/L and the fractional excretion of potassium (FeK) was 10% which spoke against hypoaldosteronism.

The serum potassium dropped to 2.8 mmol/L since day 5 of life. There was hypochloremic metabolic alkalosis with serum chloride 96 mmol/L (reference range 98–113 mmol/L). The urine biochemistry was suggestive of salt wasting and concentration disability with spot urine potassium of 8.7 mmol/L, FeK 19.9%; Fractional excretion of sodium (FeNa) 9.5%; urine chloride of 81 mmol/L, fractional excretion of chloride 11% and urine osmolality of 257 mOsm/kg. She also had hypercalciuria with urine calcium to creatinine ratio of 5.34 mmol/mmol. Meanwhile, endocrine investigations were all unremarkable except markedly elevated renin and aldosterone (Table 1). Newborn metabolic screening also excluded SW-CAH.

**Table 1:** Initial investigation results before hydrocortisone.

Investigation	Results	Normal range
Spot cortisol	481 nmol/L	101–536 nmol/L
ACTH	1.4 pmol/L	≤10.2 pmol/L
17-hydroxyprogesterone	3 nmol/L	<8 nmol/L
Androstenedione	1.1 nmol/L	0.7–10.1 nmol/L
Testosterone	<0.2 nmol/L	<0.8 nmol/L
Renin	284.88 ng/ml/h	2–35 ng/ml/h
Aldosterone	11,440 pmol/L	≤6,025 pmol/L

### 2.3 Genetic testing

Buccal swab together with parental blood samples were then sent for urgent trio Whole Exome Sequencing (WES) on day 12 of life for genes related to renal tubulopathies, fetal anomalies and aldosterone disorders. It was performed by Roche KAPA HyperExome using Illumina NextSeq550. Target mean sequencing depth was 100× and a minimum of 99% of target bases were sequenced to at least 20×. Single nucleotide variants, small insertion-deletion variants (≦15 bp) and copy number variants, which were located in exons and 10 bp of adjacent intronic sequences were targeted for curation. Exome analysis detected compound heterozygous missense variants in exon 3 of *KCNJ1* (NM_153766.3) gene: the maternally-inherited c.598A>C p.(Ser200Arg) variant and the novel paternally-inherited c.589C>T p. (Leu197Phe) variant. Both variants were classified as likely pathogenic and diagnosis of BS type II was confirmed on day 30 of life.

### 2.4 Treatment and outcome

Indomethacin was started at corrected 37 gestation weeks, stepping up the dose from 1 mg/kg/day to 3 mg/kg/day in 2 weeks. Sodium and potassium supplements were tapered off at 3 months old with normal electrolytes and renal function. Hypercalciuria improved with the latest urine calcium to creatinine ratio at 0.85 mmol/mmol. Ultrasonography of the kidneys showed medullary nephrocalcinosis. Fractional excretion of sodium and potassium reduced to 0.9% and 9.5% respectively. Serum creatinine remained normal. Serum renin and aldosterone levels also normalized. Upon follow up, she had satisfactory weight gain.

## 3 Discussion

### 3.1 Pathophysiology of BS type II

BS has an estimated incidence of 0.1 in 100,000 individuals. To date, five genes have been identified for various phenotypic presentations of BS. BS type II accounts for 19% of BS according to a systematic review (3). BS type II is an autosomal recessive disorder by the mutation of the *KCNJ1* gene. This gene encodes the apical renal outer medullary potassium channel (ROMK) which allows recycling of potassium into the tubular lumen in the TAL of the loop of Henle. The decreased recycling of potassium impairs the function of the sodium-potassium-chloride cotransporter which is responsible for sodium, potassium and chloride reabsorption, resulting in salt losing and decreased medullary interstitial hypertonicity. Thus, the water reabsorption in the collecting duct is impaired resulting in isothenuria. The impaired reabsorption of sodium chloride and potassium leads to decrease of tubular lumen positivity which is important in calcium and magnesium reabsorption. Therefore, calcium and magnesium loss in urine are features of BS type II. Hypercalciuria can lead to nephrocalcinosis which is common in BS type II (4). ROMK is also present in the macula densa in the juxta-glomerulus apparatus which, in normal function, detects the sodium chloride concentration in the distal convoluted tubule. Despite sodium chloride loss in the urine, the faulty ROMK in the macula densa fails to sense it, resulting in the release of prostaglandin and paradoxical activation of the renin-angiotensin system (RAS).

BS type II is classically presented with hyperkalemia then hypokalemia which mimics PHA type I on first presentation. One proposed mechanism of transient postnatal hyperkalemia in BS type II is the decreased Na-K-ATPase activity related to prematurity, causing a shift of potassium to the extracellular space. ROMK is also expressed in the cortical collecting duct, responsible for potassium secretion. Inactivation of its function reduces urinary potassium secretion and results in transient hyperkalemia (5). With the gradual maturation of the Na-K-ATPase activity, hyperkalemia resolves and evolves into hypokalemia. The role of potassium secretion of ROMK in the cortical collecting duct also explains the more easily corrected hypokalemia in BS type II.

### 3.2 Clinical presentations and differential diagnoses of BS type II

Antenatally, BS presents commonly with severe polyhydramnios and prematurity. Any unexplained severe polyhydramnios should raise suspicion of BS. Genetic tests should be offered or amniotic fluid for Bartter index may be used if genetic tests are not available (6).

Early recognition of BS type II remains challenging in the neonatal period. Our case initially presented like adrenal crisis. The most common cause is SW-CAH which requires targeted endocrine investigations and prompt treatments. With the availability of test results and treatment response, the list of differential diagnoses can then be narrowed down. SW-CAH in a non-virilized female neonate can be due to lipoid CAH, P450 cytochrome side chain cleavage deficiency and 3β-hydroxysteroid dehydrogenase deficiency (7). These were excluded after cortisol and ACTH came back normal. PHA type I then became the likely differential diagnosis of hyperreninemic hyperaldosteronism. However, the development of hypokalemia in our patient with high FeK and metabolic alkalosis spoke against it.

Importantly, hyperkalemia quickly transits into hypokalemia in BS type II. There are few case reports describing hyperkalemia, instead of hypokalemia in neonates with BS type II. Finer et al. reported in a case series involving 12 neonates with genetically confirmed BS type II having significant salt-wasting. The mean of the peak potassium level was 9 mmol/L and the mean of the trough sodium level at 124 mmol/L. Hyperkalemia in this group appeared on day 3 of life and normalized by the end of the first week (8). Nozu et al. also illustrated a case with hyperkalemia, hyponatremia and hyperreninemic hyperaldosteronism in the early neonatal period. This patient was also initially diagnosed as PHA until genetic study confirmed homozygous mutation of the *KCNJ1* gene (9). When hypokalemia occurs, focused investigations should be performed to evaluate its cause. In our case, there was evidence of urinary loss of potassium, normal blood pressure, metabolic alkalosis and markedly elevated serum renin and aldosterone. Differential diagnoses include congenital chloride diarrhea and pseudo-bartter syndrome which were excluded by a fractional excretion of chloride higher than 0.5% (10, 11). The presence of hypercalciuria and normal magnesium level distinguish BS from Gitelman syndrome.

We illustrate that biochemical findings can overlap between SW-CAH, PHA and BS type II in the early neonatal period due to transient hyperkalemia. Clinicians can use blood pressure, gas, serum and paired urine electrolytes to establish the diagnosis in cases of potassium disturbance (Table 2).

**Table 2:** Approach to hypokalemia with high urine potassium.

Normal or low blood pressure	Differential diagnosis
Metabolic alkalosis	
Low urine chloride	- Congenital chloride diarrhoea - Chloride deficient diet - Hypercapnea - Pseudo-bartter syndrome e.g., in cystic fibrosis
High urine chloride	- Bartter syndrome (with hypercalciuria) - Gitelman syndrome (with hypocalciuria) - Diuretic use
Metabolic acidosis	- Renal tubular acidosis - Fanconi syndrome
High blood pressure	Differential diagnosis
High renin	- Renal artery stenosis - Renal parenchymal disease - Renal tumour/pheochromcytoma
Low renin and high aldosterone	- Primary hyperaldosteronism
Low renin and low aldosterone	- Exogenous mineralocorticoid/glucocorticoid - 17 alpha hydroxylase deficiency - 17 beta hydroxylase deficiency - Liddle syndrome

### 3.3 Genetic diagnosis of BS

The European Rare Kidney Disease Reference Network Working Group (ERKNet Working Group) for Tubular Disorders recommends genetic testing for all patients with clinical suspicion of BS. BS type I to IV is autosomal recessively inherited and BS type V is X-linked recessively inherited. The detection of inactivating mutations in both alleles confirms the diagnosis of recessive forms of BS. The sensitivity and specificity of the current genetic test is 75% and 100% respectively (12).

For our case, a likely pathogenic variant of *KCNJ1* gene [c.598A>C p. (Ser200Arg)] was detected from the maternally inherited allele. This variant is absent in control population in gnomAD. However, a different variant (c.600C>G and c.600C>A)) giving rise to the same amino acid change has been reported to be pathogenic in patients with clinical features of BS (PMID: 8841184. 24400161. 29942493. ClinVar VCV001995667.2. VCV000009155.2). Experimental evidence and functional studies also suggest that p.(Ser200Arg) change affects potassium channel activity and function (PMID: 8841184. 9580661. 2440161). REVEL score is 0.926. Therefore, this variant is considered to be likely pathogenic. The *KCNJ1* gene [c.589C>T p. (Leu197Phe)] was detected from the paternally inherited allele. This variant has not been reported in human mutation databases and literature so far. REVEL score is 0.774 fulfilling a moderate score for in-silico prediction according to American College of Medical Genetics (ACMG) criteria. Both variants are absent in control populations in gnomAD. These two variants are only 3 amino acids apart and are situated in the potassium channel inwardly rectifying (Kir) cytoplasmic COOH-termini protein domain (PMID: 35463019). Our case demonstrated classical features of BS type II with specific clinical and biochemical phenotypes, caused by compound heterozygous KCNJ1 variants c.598A>C p. (Ser200Arg) and c.589C>T p. (Leu197Phe). Whole exome sequencing did not detect any other clinically significant variants, reducing the possibility of other differential diagnoses that were initially suspected.

### 3.4 Treatments

Initial treatment includes supplementation with sodium chloride and potassium chloride. Electrolyte supplements should be spread throughout the day to minimize the fluctuation of plasma level. Excessive salts will exaggerate the polyuria and lead to detrimental secondary bladder dysfunction or refluxing diseases. Therefore, judicious use of salt supplements with realistic targets can be made for patients with BS. Patients with BS type II may develop secondary nephrogenic diabetes insipidus and chronic use of sodium supplementation can aggravate hypernatremic dehydration (13). Non-Steroidal Anti-Inflammatory Drug (NSAID) is recommended for all BS to suppress cyclooxygenase and prostaglandin production, leading to decreased activation of the RAS. The ERKNet Working Group suggests that patients may develop “tolerance” to NSAID over time (12) so its use should be individualized to biochemical and clinical response. During follow up, it is crucial to monitor the growth, hydration, serum and urine biochemistry and development of nephrocalcinosis.

Our patient was started on indomethacin reaching full dosage of 3 mg/kg/day. The RAS was suppressed with improvement of serum and urinary excretion of electrolytes.

## 4 Conclusion

Clinical and biochemical features of SW-CAH and PHA type I mimic that of BS type II with salt wasting in the early neonatal period, making the diagnostic process challenging. Acute adrenal insufficiency is rare in the neonatal period. Treatment should be instituted before investigation results are available. The presence of unexplained antenatal polyhydramnios and polyuria prompts targeted investigations of renal causes of salt-losing picture. Genetic tests provide a definitive diagnosis of BS and can guide subsequent management and genetic counselling. The typical clinical features of our patient suggest the *KCNJ1*[c. 589C>T p. (Leu197Phe)] is a novel likely pathogenic variant.

## Data Availability

The raw data supporting the conclusions of this article will be made available by the authors, without undue reservation.
